# Screening of a Novel Synonymous *DNAH5* Variant in Histopathologically Confirmed Adenomyosis Cases from Turkiye

**DOI:** 10.3390/biomedicines14071435

**Published:** 2026-06-24

**Authors:** Berivan Guzelbag, Sevcan Aydin, Nimet Eser Ma, Nura Fitnat Topbas Selcuki, Engin Oral, Feyza Nur Tuncer

**Affiliations:** 1 Department of Obstetrics and Gynecology, Haseki Training and Research Hospital, University of Health Sciences Türkiye, 34265 Istanbul, Türkiye; drguzelbag@gmail.com; 2 Department of Molecular and Medical Genetics, Institute of Graduate Studies, Biruni University, 34020 Istanbul, Türkiye; 3 Department of Genetics, Graduate School of Health Sciences, Istanbul University, 34093 Istanbul, Türkiye; aydin.sevcan.20@ogr.iu.edu.tr (S.A.); nimeteser1@gmail.com (N.E.M.); 4 Department of Genetics, Aziz Sancar Institute of Experimental Medicine, Istanbul University, 34093 Istanbul, Türkiye; ftuncer@istanbul.edu.tr; 5 Department of Obstetrics and Gynecology, Şişli Hamidiye Etfal Training and Research Hospital, University of Health Sciences Türkiye, 34453 Istanbul, Türkiye; fitnat.topbas@gmail.com; 6 Department of Obstetrics and Gynecology, Norrland University Hospital, Umeå University, 90187 Umeå, Sweden

**Keywords:** adenomyosis, *DNAH5*, synonymous variant, Sanger sequencing, whole exome sequencing, ciliary dysfunction, genetic susceptibility

## Abstract

**Background/Objectives:** Adenomyosis is a common estrogen-dependent gynecological condition with a largely undefined genetic architecture. Ciliary dysfunction has been implicated in its pathogenesis, positioning genes governing ciliary structure and motility as biologically plausible candidates for investigation. The *DNAH5* gene encodes a critical component of the outer dynein arms within the ciliary axoneme, and pathogenic variants are among the most common causes of primary ciliary dyskinesia. This study aimed to systematically determine the frequency of a novel synonymous *DNAH5* variant, NM_001369.3:c.9258C>T, p.(Leu3086=), in a large, histopathologically confirmed sporadic adenomyosis cohort from Turkiye, and to evaluate its occurrence relative to population-level reference data. **Methods:** A total of 121 women with histopathologically confirmed adenomyosis following hysterectomy were enrolled. Sanger sequencing was performed under stringent quality control conditions, including primer specificity verification by NCBI BLAST and UCSC In Silico PCR. Variant frequency was compared against gnomAD v4.0 and an in-house Turkish exome database (NGS Cloud; ~30,000 sequences) using Fisher’s exact test. In silico splice site analysis was performed using SpliceAI, and variant classification followed ACMG/AMP guidelines. **Results:** The variant was detected in 63 of 121 patients (52.1%; 95% CI: 43.1–61.0%), exclusively in the heterozygous state; no homozygous carriers were identified. The variant was absent from both gnomAD v4.0 across all populations and the NGS Cloud Turkish exome database (MAF: 0.0000), yielding a frequency difference (*p* < 2.2 × 10^−16^). SpliceAI analysis predicted no significant splice site impact (all delta scores < 0.1). The variant was classified as a variant of uncertain significance (VUS; BP7, PM2_supporting). **Conclusions:** This study identifies a difference in the frequency of a novel synonymous *DNAH5* variant between a histopathologically confirmed adenomyosis cohort from Turkiye and population-level reference datasets, in which the variant was absent. Given the unphenotyped nature of the reference dataset, these findings are hypothesis-generating and do not establish a causal genetic association. Replication in independent cohorts and functional studies are warranted to elucidate the biological significance of this variant in adenomyosis susceptibility.

## 1. Introduction

Adenomyosis is a benign gynecological condition characterized by the presence of endometrial glands and stroma within the myometrium, surrounded by hypertrophic and hyperplastic smooth muscle [1,2]. Although historically regarded as a condition predominantly affecting multiparous women in their fourth and fifth decades of life, improved imaging modalities have broadened this perspective, with increasing diagnoses now reported across the reproductive years [3,4]. Clinical manifestations vary widely and include abnormal uterine bleeding, dysmenorrhea, chronic pelvic pain, and infertility [5]. Furthermore, adenomyosis is frequently comorbid with endometriosis, reported in approximately 35–79% of women with endometriosis [6]. Although frequently comorbid, the two conditions are now recognized as distinct entities with different underlying mechanisms [7,8,9]. Despite significant advances in transvaginal ultrasonography and magnetic resonance imaging, histopathological examination of hysterectomy specimens remains the gold standard for definitive diagnosis, providing the highest degree of diagnostic certainty [10,11,12].

Current understanding of adenomyosis pathogenesis centers on the invagination theory, proposing that adenomyotic lesions arise from direct invasion of endometrial basalis into the myometrium through disruption of the endometrial–myometrial interface [13,14,15]. Emerging evidence further indicates that structural abnormalities in endometrial cellular architecture play a crucial role in this pathogenic process. Khan et al. demonstrated that women with adenomyosis exhibit disruption or loss of the characteristic 9 + 2 axonemal microtubule pattern in the apical endometrium, threatening the structural integrity of the endometrial layer [14]. Importantly, this ciliary abnormality is restricted to the endometrial epithelium and is distinct from tubal ciliary dysfunction. This impairment of ciliary organization may contribute to the subfertility and adverse reproductive outcomes frequently observed in affected women [14,16].

Despite advances in understanding adenomyosis pathogenesis through molecular and cellular studies, the genetic factors contributing to disease susceptibility remain poorly characterized. Genomic characterization is increasingly recognized as essential in investigating adenomyosis and related gynecological conditions; however, the genetic architecture of this disease remains complex, involving multiple genes, gene families, and biological signaling pathways [15,17,18]. The ciliary and microtubule abnormalities documented in adenomyosis endometrium suggest that genes governing ciliary structure and function represent biologically plausible candidates for involvement in disease pathogenesis [14]. Supporting this hypothesis, variants in ciliary genes such as *DNAH11* and *CCNO* have been associated with primary ciliary dyskinesia and female infertility, establishing a broader role for ciliary dysfunction in female reproductive disorders [19]. The *DNAH5* gene encodes the axonemal dynein heavy chain 5, a critical force-generating component of the outer dynein arms essential for ciliary motility; pathogenic variants in this gene are among the most common causes of primary ciliary dyskinesia [19]. Given *DNAH5*’s fundamental role in ciliary dynamics and the well-documented ciliary abnormalities in the adenomyosis endometrium, this gene represents a compelling candidate for genetic investigation in adenomyosis susceptibility. Emerging evidence from related gynecological conditions further supports the broader genetic underpinning of adenomyosis susceptibility; functional studies in endometriosis-derived organoid models have implicated dysregulation of genes involved in genomic integrity [20], and Sanger sequencing-based SNP analyses have identified heritable disease-associated variant profiles in endometriosis within the Turkish population [21].

Although synonymous variants have traditionally been regarded as functionally neutral owing to their lack of effect on amino acid sequence, accumulating evidence demonstrates that they can significantly influence gene expression and protein function through multiple mechanisms. These include alterations in mRNA splicing efficiency, disruption of exonic splicing enhancers or silencers, changes in translation kinetics, codon usage bias affecting co-translational protein folding, and modifications in RNA secondary structure stability [22,23,24,25]. In this context, a *DNAH5* variant was initially identified through whole-exome sequencing in a family-based adenomyosis study within our group and validated by Sanger sequencing. During this validation, a novel synonymous variant, NM_001369.3:c.9258C>T, p.(Leu3086=), was incidentally observed at elevated frequency in an independent adenomyosis cohort. This variant was absent from all queried public databases, including gnomAD and ClinVar, at the time of identification.

The present study aimed to systematically determine the frequency of this novel *DNAH5* synonymous variant in a large, histopathologically confirmed sporadic adenomyosis cohort from Turkiye, and to evaluate its occurrence relative to population-level reference data from a Turkish exome database. This variant frequency characterization study was designed to assess whether this variant occurs beyond the index family in unrelated adenomyosis cases, thereby contributing to the limited genomic data available for this condition in non-European populations. To our knowledge, no published study has previously investigated *DNAH5* genetic variants in a histopathologically confirmed adenomyosis cohort of this size.

## 2. Methods

### 2.1. Patients and Clinical Assessments

Patients were retrospectively identified from hospital records based on histopathologically confirmed adenomyosis following hysterectomy at a tertiary care hospital in Istanbul, Turkiye, between January 2017 and December 2024, and subsequently invited for prospective genetic analysis. Eligible patients were identified through a review of medical records, and a total of 121 patients were included in the study. All consecutive patients who met the inclusion criteria and were available for recall were enrolled. Adenomyosis was diagnosed based on the standard histopathological criterion: the presence of endometrial glands and stroma within the myometrium. As pathology reports were generated during routine clinical care prior to study conception, pathologists were blinded to genotype status.

Inclusion criteria were: (i) women who underwent hysterectomy with histopathologically confirmed adenomyosis; (ii) availability for recall and peripheral blood sampling; and (iii) provision of written informed consent for genetic analysis. Exclusion criteria were: (i) endometriosis identified during laparotomy or laparoscopy based on surgical reports or histopathological evidence of endometriosis, to ensure a pure adenomyosis phenotype; (ii) coexistent uterine pathology, including leiomyoma or endometrial polyps, to ensure a pure adenomyosis phenotype; (iii) history of malignancy, chemotherapy, or radiotherapy, including gynecological malignancies (endometrial, cervical, or ovarian cancer), endometrial hyperplasia with atypia, and cervical intraepithelial neoplasia (CIN), which might affect genomic integrity; and (iv) known or documented history of primary ciliary dyskinesia (PCD), genetic syndromes, chronic inflammatory diseases, or autoimmune diseases based on medical records.

Clinical data were retrospectively collected from electronic medical records and surgical reports. Variables included patient age and menopausal status at the time of surgery, obstetric history (gravida, parity, abortus), and medical history (including comorbidities, previous malignancies, and systemic diseases). Menopausal status at the time of surgery was ascertained from electronic medical records and surgical reports. In cases where menopausal status was not explicitly documented, women aged 48 years or older were classified as postmenopausal, in accordance with the reported median age of natural menopause in Turkish women. Following telephone contact, patients who agreed to participate were invited to the outpatient clinic. Each participant was evaluated by the same experienced gynecologist. Prior to enrolment, surgical reports and histopathological records were reviewed to confirm the absence of endometriosis; patients were excluded if endometriotic foci were documented in operative notes or if endometriosis was identified in histopathological examination of surgical specimens. Peripheral blood samples (10 mL) were collected into ethylenediaminetetraacetic acid (EDTA) tubes by trained nursing staff, stored at 4 °C, and transported to the genetics laboratory within 24 h for DNA extraction.

### 2.2. Genetic Analyses

Genomic DNA was isolated from collected blood samples using the PureLink Genomic DNA Mini Kit (Thermo Fisher Scientific, Waltham, MA, USA) following the manufacturer’s protocol. DNA quantity and purity were assessed using a NanoDrop™ 2000 Spectrophotometer and an Invitrogen™ Qubit™ 3 Fluorometer with Qubit™ dsDNA Quantification Assay Kits (Thermo Fisher Scientific, Waltham, MA, USA); samples with A260/A280 ratios between 1.8 and 2.0 were considered acceptable for downstream analysis. Extracted DNA was stored at −80 °C until further use. The *DNAH5* reference sequence (NM_001369.3) was retrieved from the Ensembl database (https://www.ensembl.org, accessed on 20 June 2026).

Primers flanking the *DNAH5* p.(Leu3086=) variant were designed using Primer3Plus software (version 3.3.0 https://www.primer3plus.com, accessed on 20 June 2026), with full design parameters provided in Supplementary Figure S1A. The primer pair—*DNAH5*_F (5′-GAGATCCAGCTGAGGCAGAG-3′) and *DNAH5*_R (5′-TGTGTGTACTGAATTTGCATGCC-3′)—generated a 401 bp amplicon, with the sequencing read initiated from the forward primer positioned 189 bp upstream of the target variant (Supplementary Figure S1B). Amplicon specificity was confirmed using the NCBI Nucleotide BLAST (https://blast.ncbi.nlm.nih.gov/Blast.cgi, accessed on 20 June 2026) (blastn) web server (National Center for Biotechnology Information, Bethesda, MD, USA; https://blast.ncbi.nlm.nih.gov/Blast.cgi, accessed on 20 June 2026), which identified a single on-target product within *DNAH5* (Supplementary Figure S1C), and independently validated by UCSC In Silico PCR, confirming the absence of common SNPs at primer binding sites (Supplementary Figure S1D). A mismatch-tolerant, genome-wide off-target analysis was additionally performed using MFEprimer version 3.1, which identified the intended *DNAH5* amplicon as the optimal product, with potential off-target sites showing substantially different predicted amplicon sizes and melting temperatures. Variant coordinates are reported in both GRCh37 (hg19) and GRCh38 (hg38) reference assemblies. Genotype calls were assigned by direct visual inspection of the electropherograms, based on the presence of the expected secondary peak at the variant position. As an additional quality-control procedure, representative Sanger chromatograms were also manually inspected using SnapGene Viewer (version 8.2.0; Dotmatics, San Diego, CA, USA). At the c.9258C>T variant position, signal intensities corresponding to the reference and alternative nucleotides were visually evaluated to assess the reliability of heterozygous genotype calls. Representative heterozygous samples showed concurrent reference and alternative nucleotide signals with comparable peak intensities, whereas wild-type samples displayed a single predominant reference signal.

PCR amplification was performed using MyTaq™ Red DNA Polymerase (Bioline, London, UK) according to the manufacturer’s protocol. Each 25 µL reaction contained 12.5 µL MyTaq Red Mix, 1 µL of each primer (10 µM), 50–100 ng genomic DNA, and nuclease-free water. Thermal cycling was carried out on a Bio-Rad T100 thermal cycler with the following conditions: initial denaturation at 95 °C for 3 min, followed by 35 cycles of denaturation at 95 °C for 30 s, annealing at 60 °C for 30 s, and extension at 72 °C for 30 s, with a final extension at 72 °C for 5 min. Amplification products were verified by 2% agarose gel electrophoresis with ethidium bromide staining and visualized under ultraviolet illumination. Sanger sequencing was performed by. Macrogen, Inc., Seoul, South Korea; sequencing data were obtained in .ab1 format and analyzed using CLC Main Workbench 6.5. Approximately 5% of samples yielded suboptimal sequencing results due to background noise; these samples were re-sequenced, and those with persistent results underwent repeated PCR amplification followed by re-sequencing.

To characterize the population-level frequency of the *DNAH5* c.9258C>T variant, allele frequencies were assessed against two reference datasets: the Genome Aggregation Database (gnomAD) v4.0 (https://gnomad.broadinstitute.org/, accessed on 20 June 2026) and the NGS Cloud in-house database (https://search.ngscloud.com/, accessed on 20 June 2026), comprising approximately 30,000 whole-exome sequences from individuals in Turkiye with varying clinical indications. The queried locus is not flagged for low complexity or poor coverage within the gnomAD v4.0 database. This locus is well covered in gnomAD, with the majority of individuals sequenced at high depth at this position, indicating that it is reliably callable in short-read sequencing data. Prior to reporting variant absence, NGS Cloud applies stringent quality thresholds, including a minimum read depth of 30×, a variant allele fraction greater than 0.25, a base quality score above 30, and the absence of significant strand bias [18]. These datasets represent unphenotyped population frequency references and were not utilized as case–control comparators. Splice site impact of the variant was assessed using the SpliceAI Lookup tool (Broad Institute; https://spliceailookup.broadinstitute.org, accessed on 20 June 2026), which implements a deep learning model for splice site prediction [26]. Predictions were generated using the GRCh38 (hg38) reference genome with GENCODE transcript annotation under default parameters.

### 2.3. Statistical Analysis

Statistical analyses were performed using R software version 4.3.3 (R Foundation for Statistical Computing, Vienna, Austria). Descriptive statistics were applied to clinical variables; continuous variables were summarized as mean ± standard deviation (SD) and categorical variables as frequencies and percentages. Demographic and clinical characteristics were compared between variant carriers and non-carriers using the independent samples *t*-test for continuous variables and the chi-square test for categorical variables. Minor allele frequency (MAF) was calculated as the number of variant alleles divided by the total number of alleles (2n). The 95% confidence interval (CI) for the carrier proportion was calculated using the Wilson score method. The frequency of the *DNAH5* c.9258C>T variant in the adenomyosis cohort was compared with the NGS Cloud population frequency reference dataset using Fisher’s exact test. All tests were two-tailed with a significance threshold of *p* < 0.05; no correction for multiple comparisons was applied, as a single genetic variant was evaluated; in single-variant studies, the risk of type I error inflation due to multiple testing is not applicable.

## 3. Results

### 3.1. Clinical Characteristics of the Study Cohort

A total of 121 women with histopathologically confirmed adenomyosis were included in the final analysis. Patients were classified as variant carriers (*n* = 63) or non-carriers (*n* = 58) based on Sanger sequencing results. Demographic and clinical characteristics, stratified by variant carrier status, are summarized in Table 1.

The mean age at surgery was 49.3 ± 8.4 years, with the majority of patients in the 46–55 years age group (62.8%), followed by the 36–45 years group (19.8%) and those older than 55 years (17.4%). Most patients were postmenopausal at the time of surgery (62.0%). The cohort was characterized by high parity, with a mean parity of 3.6 ± 2.3 and only 4 patients (3.3%) being nulliparous. Abnormal uterine bleeding was the predominant surgical indication, reported in 108 patients (89.3%).

No statistically significant differences were observed between variant carriers (*n* = 63) and non-carriers (*n* = 58) in any of the assessed clinical variables, including age at surgery (51.0 ± 8.1 vs. 47.5 ± 5.9 years; *p* = 0.108), menopausal status (*p* = 0.140), gravida (4.5 ± 2.6 vs. 4.1 ± 2.3; *p* = 0.892), parity (3.7 ± 2.3 vs. 3.5 ± 2.1; *p* = 0.943), and rate of abnormal uterine bleeding (90.5% vs. 87.9%; *p* = 0.658).

### 3.2. Genetic Findings

**Figure 1:**
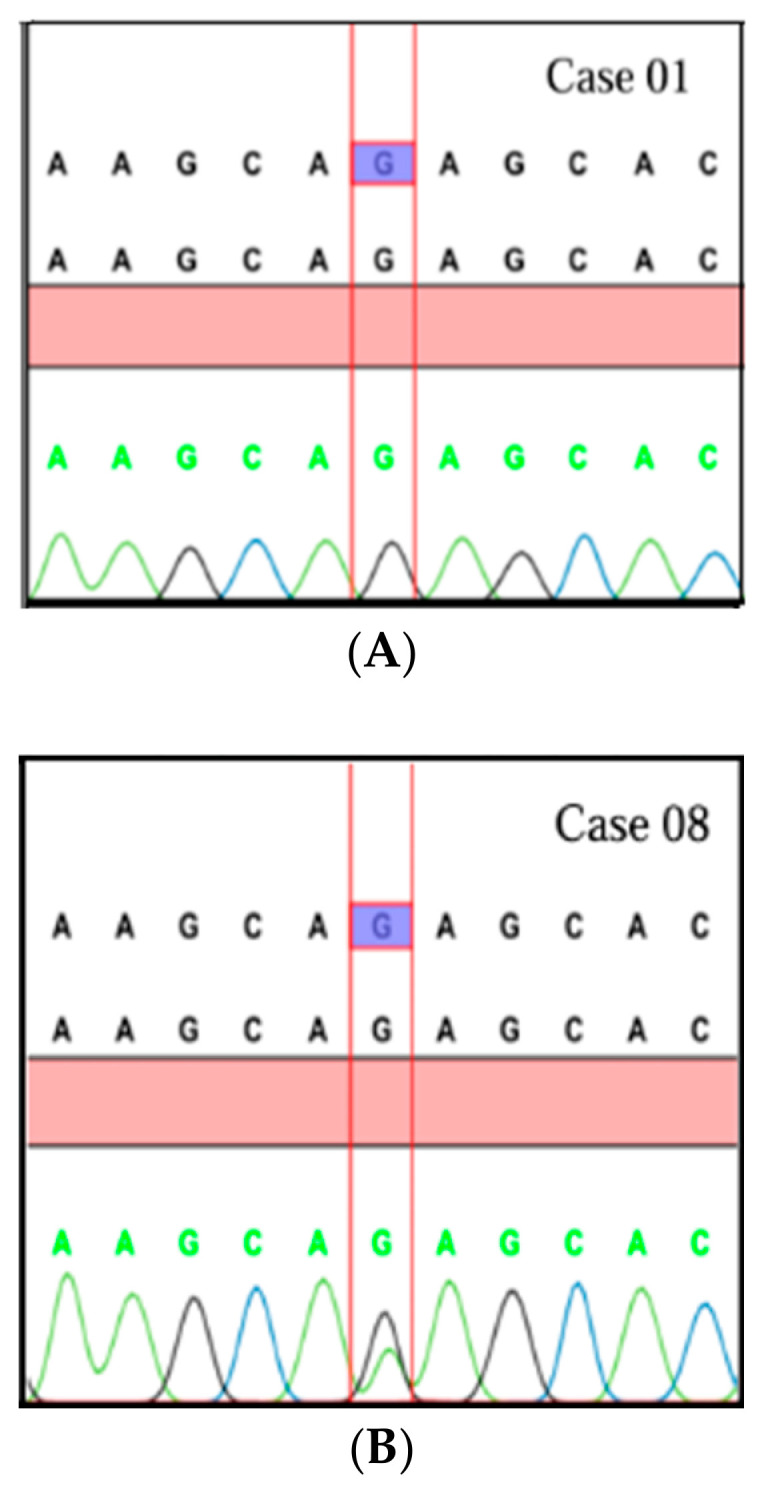
Representative Sanger sequencing chromatograms of the *DNAH5* c.9258C>T variant, shown in the reverse (antisense) orientation. (**A**) Homozygous reference genotype (CC), Case 01: a single G (black) peak is observed at the variant position, corresponding to the reference C on the sense strand. (**B**) Heterozygous genotype (CT), Case 08: both G (black) and A(green) peaks are present at the variant position (indicated by the red vertical line), corresponding to the C>T change on the sense strand, confirming the heterozygous state. Peak color coding is as follows: A, green; C, blue; G, black; and T, red. The purple box indicates the reference nucleotide (G) at the corresponding position on the reverse strand. Sequence analyses were performed using CLC Main Workbench 6.5 (QIAGEN, Aarhus, Denmark).

The novel synonymous *DNAH5* variant NM_001369.3:c.9258C>T, p.(Leu3086=) was detected in 63 of 121 patients (52.1%; 95% CI: 43.1–61.0%), exclusively in the heterozygous state; no homozygous carriers were identified. This corresponds to a minor allele frequency (MAF) of 26.0% (63 of 242 alleles) within the adenomyosis cohort. The variant was absent from both the gnomAD v4.0 database across all populations (MAF: 0.0000) and the NGS Cloud in-house Turkish exome database (MAF: 0.0000), and its frequency in the adenomyosis cohort was higher than in the population frequency reference dataset (63/121 vs. 0/30,000; *p* < 2.2 × 10^−16^). Representative Sanger sequencing chromatograms for wildtype (CC) and heterozygous carrier (CT) genotypes are presented in Figure 1; chromatograms for all 121 patients are provided in Supplementary Figure S1E. In silico splice site analysis using SpliceAI yielded delta scores below 0.1 for all predicted splice sites. Variant classification according to ACMG guidelines yielded a classification of variant of uncertain significance (VUS). The variant was classified as VUS based on ACMG/AMP evidence codes BP7 (synonymous variant with no predicted splicing impact) and PM2_supporting (absent from population-level reference databases). The variant was not present in ClinVar or dbSNP at the time of analysis. Variant characteristics are summarized in Table 2.

## 4. Discussion

This study systematically characterized the frequency of a novel synonymous *DNAH5* variant, NM_001369.3:c.9258C>T, p.(Leu3086=), in a large, histopathologically confirmed sporadic adenomyosis cohort from Turkiye. The principal finding was a carrier frequency of 52.1% (95% CI: 43.1–61.0%) in 121 adenomyosis patients, contrasting with a complete absence of this variant in approximately 30,000 whole-exome sequences from an unphenotyped Turkish population reference dataset. This frequency difference represents a hypothesis-generating observation; given the unphenotyped nature of the reference dataset, it does not constitute evidence of a causal genetic association.

The demographic and clinical characteristics of our cohort are consistent with established literature on adenomyosis epidemiology. The mean age of 49.3 ± 8.4 years, with the predominance of patients in the 46–55 age group (62.8%), reflects the typical presentation of adenomyosis during the late reproductive and perimenopausal periods, when prolonged estrogen exposure and progressive symptom severity often culminate in surgical intervention [27,28]. The high parity observed in our cohort (mean 3.6 ± 2.3), with only 3.3% nulliparous patients, is consistent with the well-established relationship between multiparity and adenomyosis susceptibility, likely mediated through mechanical disruption of the myometrial junctional zone during repeated pregnancies [29,30]. Abnormal uterine bleeding was the predominant surgical indication (89.3%), in keeping with the pathophysiology of adenomyosis, wherein ectopic endometrial tissue within the myometrium increases endometrial surface area and disrupts uterine contractility [31,32]. It should be noted that, as this cohort comprised exclusively hysterectomy-based, histologically confirmed cases, the findings may not be generalizable to younger women, imaging-diagnosed adenomyosis, or milder disease phenotypes.

The biological rationale for investigating *DNAH5* in adenomyosis is supported by the ciliary abnormalities documented in the adenomyosis endometrium and by the established role of DNAH5 in ciliary motility [14,19]. In this context, the identification of this variant at elevated frequency in a histopathologically confirmed adenomyosis cohort, in conjunction with these documented ciliary abnormalities, provides a biologically plausible basis for further investigation.

Although the observed variant is synonymous, this does not preclude functional relevance, as synonymous variants may influence gene expression through several previously described mechanisms [22,23,24,25]. In the present study, SpliceAI in silico analysis predicted no significant impact on canonical splice sites, with all delta scores below 0.1. While this argues against a splicing-mediated mechanism, other functional consequences—such as altered codon usage or mRNA stability—cannot be excluded and require experimental validation. A notable observation in our cohort was the complete absence of homozygous carriers; all 63 variant-positive individuals harbored the variant exclusively in the heterozygous state. The exclusive detection of the variant in the heterozygous state, with no homozygous individuals identified, is an observation that warrants further investigation, as the biological basis of this distribution remains unclear. Although the genotype distribution deviated from Hardy–Weinberg equilibrium, such analyses are conventionally applied to population-based rather than disease-selected cohorts, and this departure is therefore not unexpected in a histopathologically defined adenomyosis cohort. The specificity of the amplicon, confirmed by NCBI BLAST and UCSC In Silico PCR, reduces the likelihood that this distribution reflects a technical artifact of paralogous co-amplification; nonetheless, the biological basis of this distribution remains to be clarified.

Several methodological strengths of this study merit consideration. All 121 cases were histopathologically confirmed following hysterectomy, providing the highest available degree of diagnostic certainty and a well-defined disease phenotype. Sanger sequencing was performed under stringent quality control conditions, including primer optimization, amplicon specificity verification by NCBI BLAST and UCSC In Silico PCR, and independent population frequency validation against both gnomAD and the NGS Cloud in-house Turkish exome database. Furthermore, the generally comparable demographic and clinical characteristics of variant carriers and non-carriers—including age, menopausal status, parity, and rate of abnormal uterine bleeding—are consistent with the observed frequency difference not being driven primarily by clinical selection bias within the cohort.

Nonetheless, several limitations must be acknowledged. The most critical is the absence of a phenotyped control group of women confirmed to be free of adenomyosis; the NGS Cloud reference dataset, while large, is unphenotyped and may include individuals with undiagnosed adenomyosis, given its reported prevalence of 10–35% in hysterectomy specimens [9]. Should undiagnosed adenomyosis cases be present within the reference dataset, the resulting bias would be directed toward the null, suggesting that the observed frequency difference may represent a conservative estimate. Additionally, individuals in this database were sequenced for heterogeneous clinical indications, introducing potential ascertainment bias. As this was a single-center study conducted in a Turkish population, generalizability to other populations remains limited. The hysterectomy-based recruitment strategy introduces selection bias toward more severe disease phenotypes, and the findings cannot be extrapolated to younger women, imaging-diagnosed adenomyosis, or conservatively managed cases. The possibility of occult or asymptomatic endometriosis not documented in operative or histopathological records cannot be fully excluded, as peritoneal endometriotic implants may be missed in the absence of dedicated laparoscopic evaluation. Detailed histopathological subtyping, including focal versus diffuse classification and depth of myometrial invasion, was not systematically recorded in routine pathology reports and therefore could not be assessed. In addition, standardized symptom severity scales (e.g., validated pain or bleeding scores) were not available from retrospective records, and no a priori power analysis was performed, as this was a frequency-characterization study rather than a case–control association study. Consequently, the absence of significant differences between carriers and non-carriers should be interpreted with caution, as it may reflect limited statistical power (type II error) rather than a true absence of genotype–phenotype association. Moreover, this study was not specifically powered for genotype–phenotype comparisons, several clinical variables such as abnormal uterine bleeding were recorded retrospectively as present or absent, without standardized severity assessment, and detailed pathological subtyping was not available from the retrospective records; consequently, clinically meaningful genotype–phenotype associations cannot be excluded and should be investigated in larger, prospectively characterized cohorts. Geographic origin data were not collected; therefore, founder effect or population stratification could not be formally assessed, although the diverse demographic composition of Istanbul—a city with significant internal migration from all regions of Turkey—suggests that our cohort likely represents a heterogeneous Turkish population. Pre-operative hormonal medication history, including levonorgestrel-releasing intrauterine devices, GnRH agonists, and oral contraceptives, could not be reliably ascertained due to the retrospective nature of patient identification and potential recall bias, as many patients underwent hysterectomy several years prior to study enrolment. Independent orthogonal validation of the variant, such as targeted next-generation sequencing or droplet digital PCR, was not performed and represents a limitation of the current study. Although primer specificity was confirmed in silico by NCBI BLAST and UCSC In Silico PCR, reducing the likelihood of paralogous co-amplification, confirmation by an independent experimental method remains warranted in future work. In addition, as variant frequencies were derived from Sanger sequencing in the adenomyosis cohort and compared with exome-based population databases, platform-specific differences in detection sensitivity cannot be entirely excluded, although the variant position was well covered in gnomAD and was initially identified through exome sequencing. Finally, given the exploratory nature and sample size of this study, the possibility of a chance finding cannot be excluded.

These findings provide a foundation for future investigations into the potential role of *DNAH5* variation in adenomyosis. Replication in larger, independent cohorts from diverse ethnic populations is essential to determine whether the observed frequency difference is population-specific or represents a broader signal. A well-designed case–control study incorporating women confirmed to be free of adenomyosis—ideally through histopathological examination—would enable formal assessment of variant–disease association. Functional studies examining the effects of the c.9258C>T variant on *DNAH5* expression, mRNA stability, codon usage, and protein function are needed to clarify its biological significance. Investigation of ciliary morphology and motility in endometrial tissue from variant carriers versus non-carriers would provide mechanistic insights into the potential link between this variant and the ciliary abnormalities documented in adenomyosis. Collectively, such studies are essential to determine whether this variant represents a biologically meaningful signal or a population-specific neutral variant, and to advance our understanding of the genetic architecture underlying adenomyosis.

## 5. Conclusions

This study reports a difference in the frequency of a novel synonymous *DNAH5* variant, NM_001369.3:c.9258C>T, p.(Leu3086=), in a large, histopathologically confirmed adenomyosis cohort from Turkiye, with a carrier frequency of 52.1% contrasting with its absence in approximately 30,000 population-level exomes. All variant-positive individuals were exclusively heterozygous, and no significant differences in clinical characteristics were observed between carriers and non-carriers. Given the unphenotyped nature of the reference dataset, these findings should be considered hypothesis-generating and do not establish a causal genetic association. Confirmation of this observation requires replication in well-powered, prospective case–control studies with histopathologically confirmed adenomyosis-free controls, complemented by functional studies to elucidate the biological significance of this variant.

## Figures and Tables

**Table 1:** Demographic and Clinical Characteristics of the Adenomyosis Cohort by Genotype.

Characteristic	Total (*n* = 121)	Carriers (*n* = 63)	Non-Carriers (*n* = 58)	*p*-Value
Age at surgery (years), mean ± SD	49.3 ± 8.4	51.0 ± 8.1	47.5 ± 5.9	0.108
Age distribution, *n* (%)				
36–45 years	24 (19.8)	10 (15.9)	14 (24.1)	
46–55 years	76 (62.8)	40 (63.5)	36 (62.1)	
>55 years	21 (17.4)	13 (20.6)	8 (13.8)	
Menopausal status, *n* (%)				0.140
Premenopausal	46 (38.0)	19 (30.2)	27 (46.6)	
Postmenopausal	75 (62.0)	44 (69.8)	31 (53.4)	
Obstetric History				
Gravida, mean ± SD	4.3 ± 2.5	4.5 ± 2.6	4.1 ± 2.3	0.892
Parity, mean ± SD	3.6 ± 2.3	3.7 ± 2.3	3.5 ± 2.1	0.943
Surgical Indication				
Abnormal uterine bleeding, *n* (%)	108 (89.3)	57 (90.5)	51 (87.9)	0.658

SD: standard deviation. *p*-values were calculated using independent *t*-test for continuous variables and chi-square test for categorical variables.

**Table 2:** Characteristics of the *DNAH5* c.9258C>T, p.(Leu3086=) Variant.

Gene (Transcript)	Location GRCh37 (hg19)	Location GRCh38 (hg38)	cDNA Change	Protein Change	Variant Type	Exon	ACMG	ClinVar	dbSNP	NGS Cloud MAF/gnomAD MAF
*DNAH5* (NM_001369.3)	chr5:13776663	chr5:13776554	c.9258C>T	p.(Leu3086=)	Synonymous	55	VUS	NR	NR	0.0000/0.0000

NR: not reported; ACMG: American College of Medical Genetics and Genomics; VUS: variant of uncertain significance; MAF: minor allele frequency.

## Data Availability

The data that support the findings of this study are available from the corresponding author upon reasonable request, subject to ethical approval and patient confidentiality requirements.

## Supplementary Materials

The following supporting information can be downloaded at https://www.mdpi.com/article/10.3390/biomedicines14071435/s1, Figure S1A: Primer design parameters for the *DNAH5* p.(Leu3086=) variant using Primer3Plus software. Figure S1B: Schematic representation of the *DNAH5* amplicon showing primer positions and target variant location. Figure S1C: NCBI BLAST analysis confirming amplicon specificity. Figure S1D: UCSC In Silico PCR analysis confirming primer specificity and absence of common SNPs at primer binding sites. Figure S1E: Sanger sequencing chromatograms of all 121 patients with histopathologically confirmed adenomyosis.
