# Supplementary material for: Screening of a Novel Synonymous DNAH5 Variant in Histopathologically Confirmed Adenomyosis Cases from Turkiye
Source: Biomedicines. 2026 Jun 24;14(7):1435. doi: 10.3390/biomedicines14071435 (PMC13404112; doi:10.3390/biomedicines14071435)
Supplement: Supplementary file 1 [file biomedicines-14-01435-s001.zip › biomedicines-4295584 -Supplementary Figure S1.pdf]

Supplementary Figure S1.

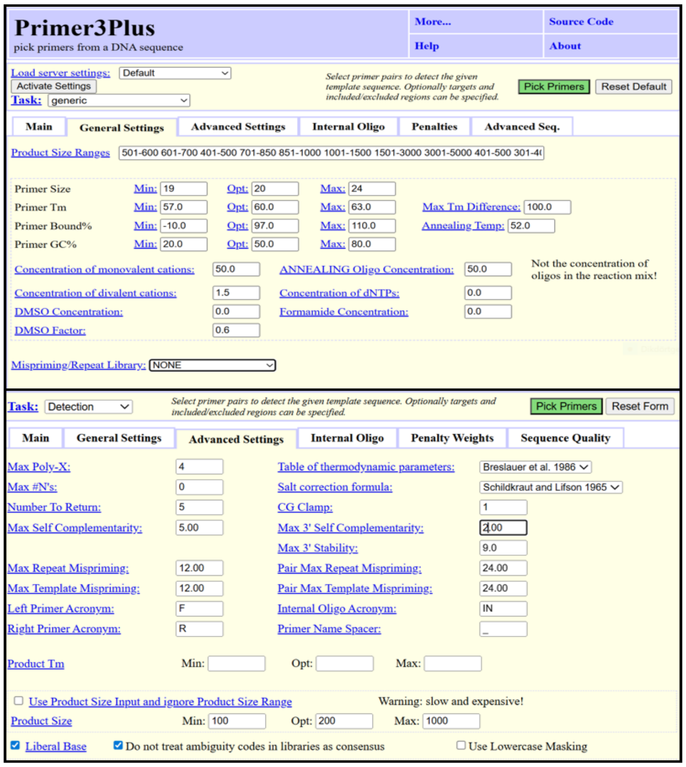

Figure S1A. Primer design parameters for the *DNAH5* p.(Leu3086=) variant. Primer pairs were designed using Primer3Plus software (version 3.3.0; https://www.primer3plus.com, accessed on 20 June 2026) following standard primer design criteria, including evaluation of potential secondary structures. Full design parameters are shown.

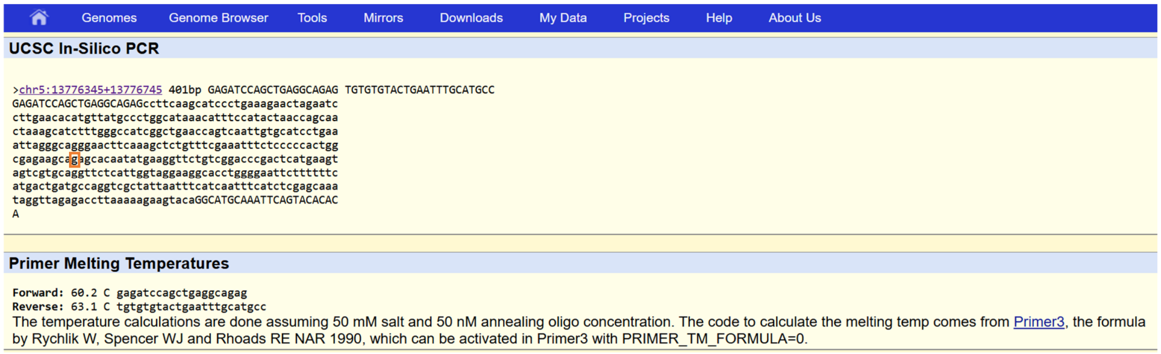

Figure S1B. Schematic representation of the *DNAH5* amplicon. The primer pair *DNAH5*_F (5′-GAGATCCAGCTGAGGCAGAG-3′) and *DNAH5*_R (5′-TGTGTGTACTGAATTTGCATGCC-3′) generated a 401 bp amplicon. Sequencing was initiated from the forward primer, positioned 189 bp upstream of the target variant. The red square indicates the position of the *DNAH5* c.9258C>T, p.(Leu3086=) variant.

Primer pair 1

	Sequence (5'->3')	Length	Tm	GC%	Self complementarity	Self 3' complementarity
**Forward primer**	GAGATCCAGCTGAGGCAGAG	20	59.61	60.00	6.00	1.00
**Reverse primer**	TGTGTGTACTGAATTTGCATGCC	23	60.31	43.48	6.00	4.00

Products on target templates

>NC_000005.10 Homo sapiens chromosome 5, GRCh38.p14 Primary Assembly

```
product length = 401
Features associated with this product:
   dynein axonemal heavy chain 5 isoform x1

   dynein axonemal heavy chain 5

Forward primer  1         GAGATCCAGCTGAGGCAGAG  20
Template        13776345  ....................  13776364

Reverse primer  1         TGTGTGTACTGAATTTGCATGCC  23
Template        13776745  .......................  13776723
```

Figure S1C. NCBI BLAST analysis confirming amplicon specificity. Primer efficiency and specificity were verified using the National Center for Biotechnology Information Basic Local Alignment Search Tool (NCBI BLAST), which identified a single on-target amplicon of 401 bp within *DNAH5*, confirming the absence of off-target amplification.

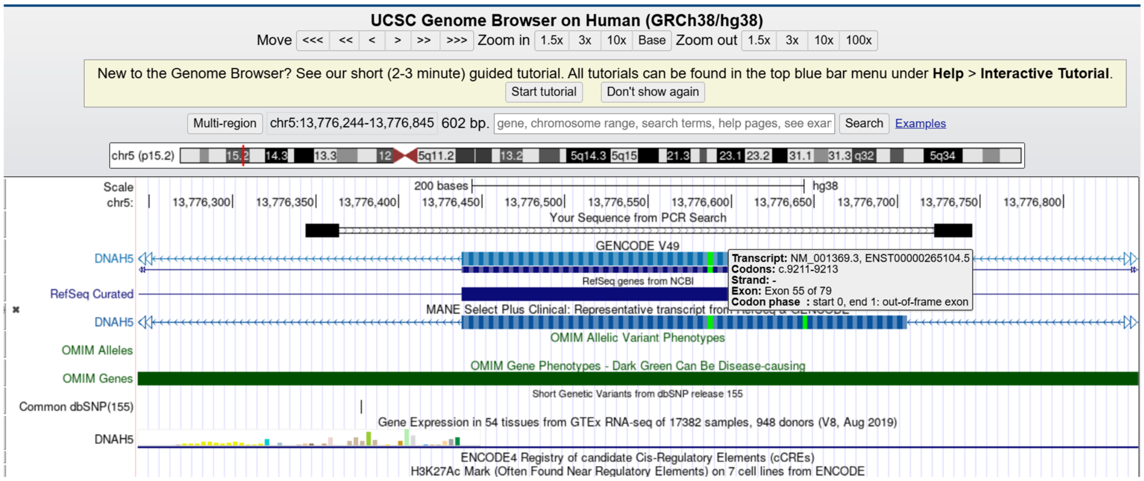

Figure S1D. UCSC In-Silico PCR analysis confirming primer specificity. A second independent quality check was performed using the UCSC In-Silico PCR tool, which confirmed primer specificity and verified the absence of common single-nucleotide polymorphisms (SNPs) at primer binding sites.

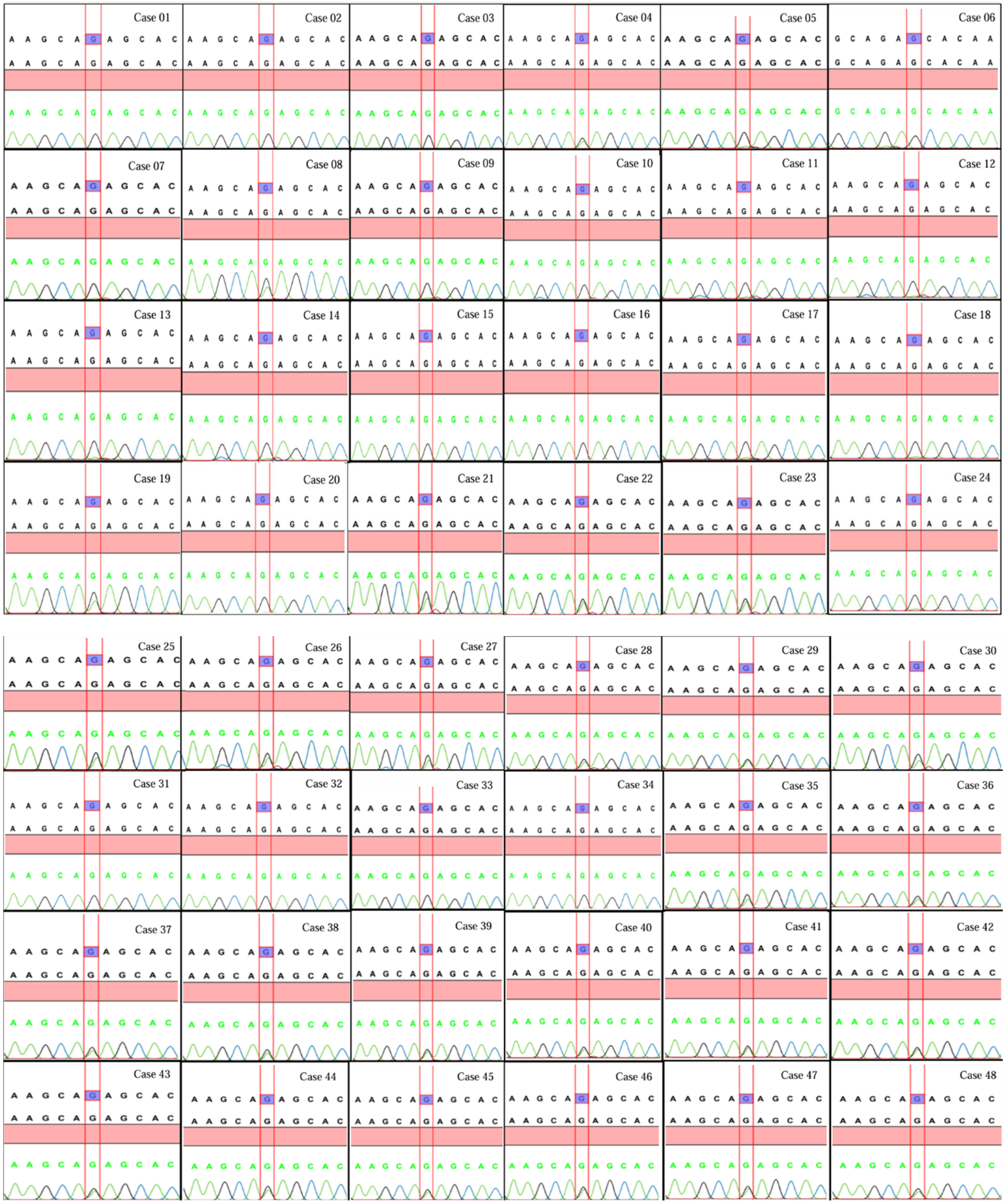
Case 01
Case 02
Case 03
Case 04
Case 05
Case 06
Case 07
Case 08
Case 09
Case 10
Case 11
Case 12
Case 13
Case 14
Case 15
Case 16
Case 17
Case 18
Case 19
Case 20
Case 21
Case 22
Case 23
Case 24
Case 25
Case 26
Case 27
Case 28
Case 29
Case 30
Case 31
Case 32
Case 33
Case 34
Case 35
Case 36
Case 37
Case 38
Case 39
Case 40
Case 41
Case 42
Case 43
Case 44
Case 45
Case 46
Case 47
Case 48

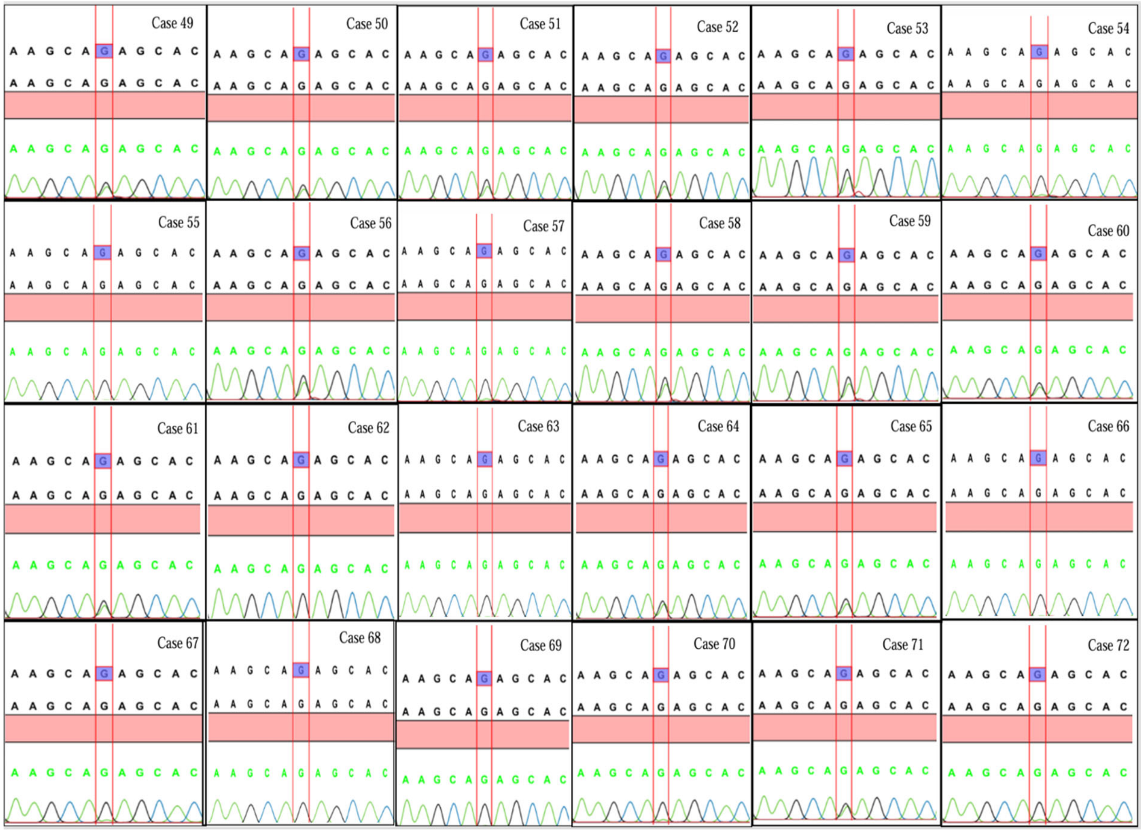
Case 49
Case 50
Case 51
Case 52
Case 53
Case 54
Case 55
Case 56
Case 57
Case 58
Case 59
Case 60
Case 61
Case 62
Case 63
Case 64
Case 65
Case 66
Case 67
Case 68
Case 69
Case 70
Case 71
Case 72

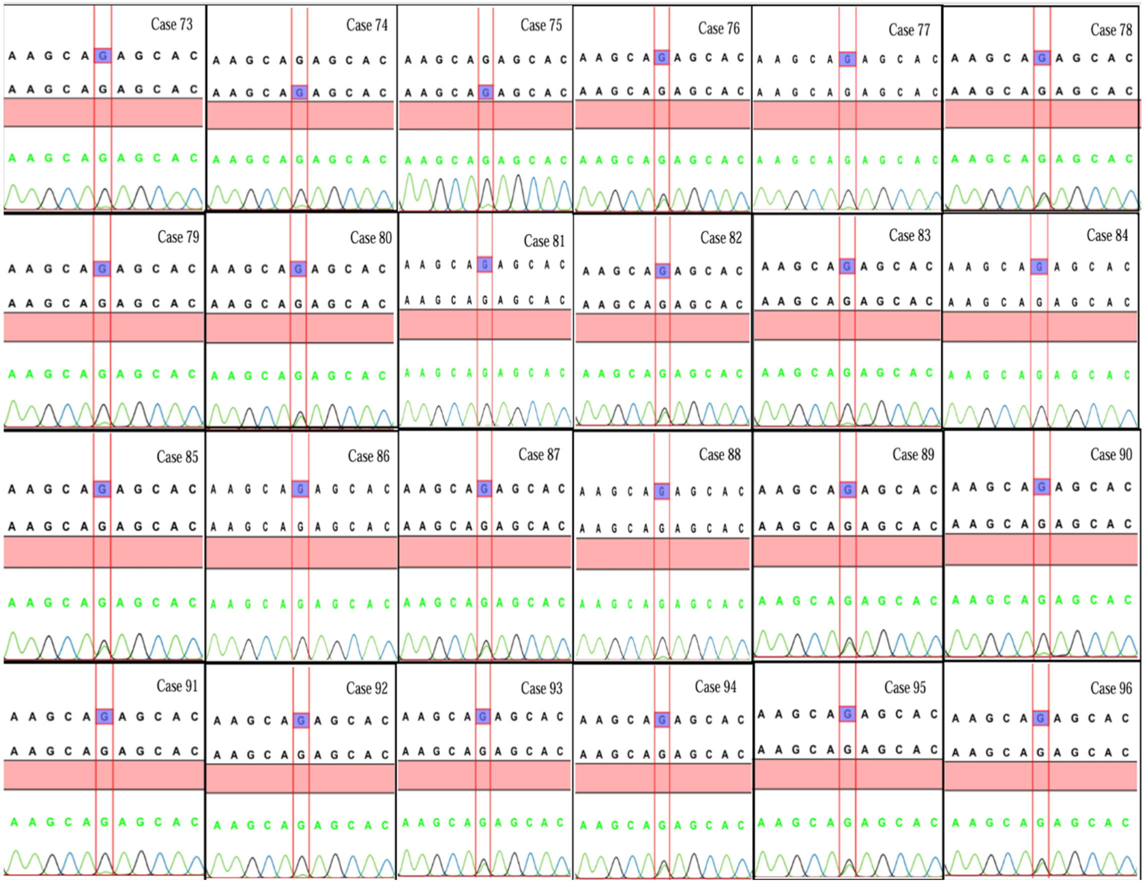
Case 73
Case 74
Case 75
Case 76
Case 77
Case 78
Case 79
Case 80
Case 81
Case 82
Case 83
Case 84
Case 85
Case 86
Case 87
Case 88
Case 89
Case 90
Case 91
Case 92
Case 93
Case 94
Case 95
Case 96

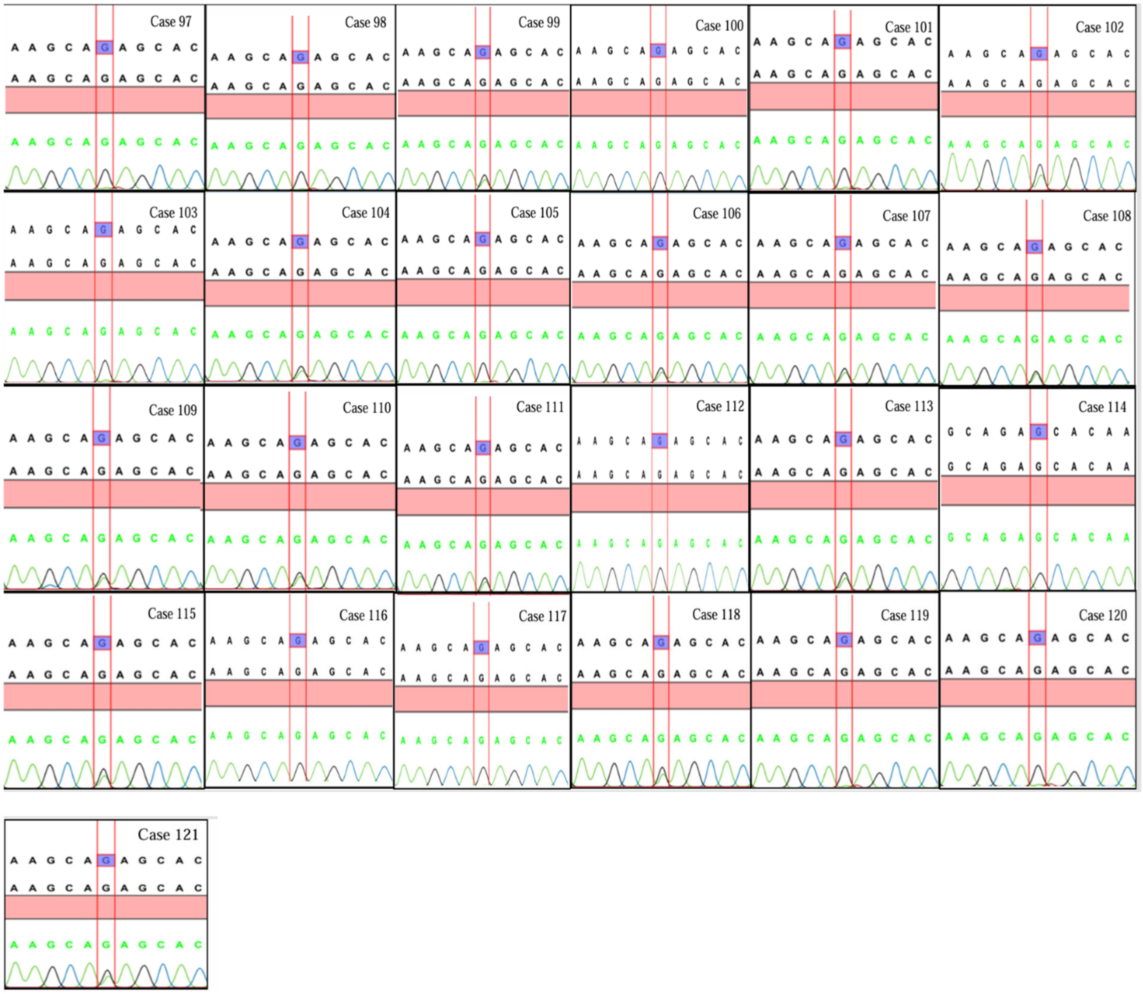

Figure S1E. Sanger sequencing chromatograms of all 121 patients with histopathologically confirmed adenomyosis, analyzed using CLC Main Workbench 6.5 (QIAGEN, Aarhus, Denmark. No homozygous carrier (TT) was identified in the cohort. Of the 121 patients, 63 exhibited the heterozygous genotype (CT) and 58 were wildtype (CC) for the *DNAH5* c.9258C>T, p.(Leu3086=) variant.
